# Determinants of Weight Loss prior to Diagnosis in Inflammatory Bowel Disease: A Retrospective Observational Study

**DOI:** 10.1155/2014/762191

**Published:** 2014-11-23

**Authors:** Yasser Elsherif, Christopher Alexakis, Michael Mendall

**Affiliations:** Gastroenterology Department, Croydon University Hospital, London Road, Croydon, London CR7 7YE, UK

## Abstract

*Aims*. To identify prevalence, severity, and environmental determinants of weight loss in inflammatory bowel disease (IBD) patients just prior to time of formal diagnosis. *Methodology*. IBD patients attending outpatient clinic were questioned about weight loss prior to diagnosis and other environmental and demographic variables. The percentage BMI loss was calculated for each subject and factors associated with weight loss were determined. *Results*. Four hundred and ninety-four subjects were recruited (237 cases of Crohn's disease (CD) and 257 cases of ulcerative colitis (UC)). Overall, 57% of subjects with CD and 51% of subjects with UC experienced significant weight loss prior to diagnosis (>5% BMI loss). Younger age at diagnosis and history of previous IBD surgery were significantly associated with both lower BMI at diagnosis and increased weight loss prior to diagnosis. In CD patients, increasing age at diagnosis was inversely associated with weight loss prior to diagnosis. Ileal disease was a risk factor of weight loss, whereas prior appendectomy was associated with reduced risk of weight loss. *Conclusions*. Weight loss is a significant problem for many IBD patients at presentation, especially in younger age and CD with ileal involvement. Appendectomy is associated with diminished weight loss.

## 1. Introduction

Disease associated malnutrition is frequent health burden and can significantly affect both disease outcome and patient's quality of life. The association between malnutrition and increased morbidity is well established in chronic diseases and the elderly [1]. Inflammatory bowel disease (IBD) represents a significant portion of a gastroenterologist's workload. Malnutrition is observed in both ulcerative colitis (UC) and Crohn's disease (CD) and in both active and quiescent periods of the diseases [2]. In paediatric patients with IBD, recent weight loss forms one of the trials of clinical manifestations and a cornerstone for the diagnosis of CD [3]. Additionally, weight loss which results from the underlying physiological disturbance associated with the disease could be regarded as a marker of disease activity. Weight loss at presentation in adult patients with IBD has not been fully studied. Similarly, the role of environmental and nonenvironmental factors that influence weight loss in this patient group is still unclear. In order to provide evidence base for understanding the epidemiology of undernutrition and weight loss in IBD patients, a retrospective observational study was undertaken for the first time to identify prevalence and severity of weight loss in IBD patients just prior to time of formal diagnosis and describe the disease and environmental variables that are associated with this.

## 2. Material and Methods

Consecutive patients with established IBD attending gastroenterology outpatient clinics at two sites at St George's Hospital, a large London teaching hospital, and Croydon University Hospital, a similar sized district general hospital in close proximity of St George's, between 2005 and 2011, were enrolled into the study. All subjects had had (1) one or more symptoms of diarrhoea, rectal bleeding, abdominal pain, or fever; (2) occurrence of symptoms on more than one occasion or continuously for at least 6 weeks; (3) objective evidence of inflammation on radiologic, endoscopic, or histological criteria; and/or (4) surgery for CD or UC in the past with or without ongoing evidence of inflammation.

Cases were assigned to CD or UC on the basis of clinical, endoscopic, histopathological, and radiological criteria. Where the diagnosis of CD or UC was unclear, subjects were assigned to UC or CD by a panel of investigators. In 21 cases, the panel felt that the diagnosis was genuinely indeterminate, and these were excluded from further analysis. Subjects with CD were classified according to the Montreal Classification. The site and disease behaviour for subjects with CD were recorded, but in some cases this was not possible due to lack of available data. Data for the extent of UC was not uniformly available as it was not routine clinical practice to use this classification for UC.

Patients were provided with a detailed standardised questionnaire which they completed with the guidance of the clinic doctor. Extra information required was gathered from the clinical case notes where appropriate. The questionnaire examined various aspects of their demographics, lifestyle, ethnic group, and so forth. Detailed clinical aspects of their disease were enquired including age at diagnosis, location of disease (ileal, ileocolonic, colonic, and others), disease character (inflammatory, stricturing, and penetrating), and history of appendectomy and surgical history. Maximum adult prediagnosis weight was recorded (self-reported), together with weight at diagnosis (self-reported and from clinical notes), as well as current weight (taken at time of questionnaire). We have previously described the validity of self-reported historical weight [4]. From a subset of 102 subjects, recalled weight was compared with actual weight measured in the clinic up to 5 years earlier. There was an excellent correlation (*r* = 0.94).

Statistical analysis was performed using StatView (Abacus Corporation, Baltimore, MD, USA). Change in BMI prior to diagnosis was defined as follows: (maximum self-reported weight prediagnosis (kg) minus measured weight at diagnosis (kg))/height (m)². A further variable of % BMI loss prior to diagnosis was generated for each patient as follows: (change in BMI prior to diagnosis/maximum prediagnosis BMI) × 100. The distribution of percentage in change in BMI was approximately normally distributed (skewness 0.99, Kurtosis 1.37), so analysis was performed using multiple regression. This was performed adjusting for various characteristics and demographic factors including age at diagnosis, sex, smoking, history of appendectomy, and site of disease. Where data for any particular variable was not available or missing, this was coded for as unknown. Fisher's exact test or student's *t*-test was used, respectively, to calculate significant differences between the proportions and means of the various subgroups analysed.

*Ethical Considerations*. Ethical approval was granted by London and Surrey Borders Ethics Committee in 2004 and subjects gave consent to participate in this study.

## 3. Results

### 3.1. Characteristics of Subjects

A total of 494 patients with IBD were recruited (257 UC, 237 CD).  Table 1 shows the disease characteristics of the study population. As would be expected in patients with CD as compared to those with UC, age at diagnosis was younger, there were a higher proportion of smokers, and there was a significantly increased number of subjects with a history of appendectomy as well as IBD related surgery, as in Table 1.

### 3.2. Univariate Analysis of Subject Characteristics and BMI at Diagnosis of Subjects with CD and UC

Table 2 shows mean BMI at diagnosis as well as percentage of BMI loss prior to diagnosis in UC and CD groups. Mean BMI at diagnosis was 23.97 kg/m² and 23.68 kg/m² for UC and CD, respectively. In both CD and UC groups, younger age at diagnosis was significantly associated with underweight defined as BMI less than 18, 68.4% versus 2.5% and 42.9% versus 3.6% for Montreal A1 versus A3 of CD and UC subjects, respectively. Current smokers had lower BMI at diagnosis, and 21.1% and 12.4% of CD and UC subjects had BMI less than 18 compared to 10.9% and 8.7% of nonsmokers. None of the other characteristics were associated with being underweight at diagnosis.

### 3.3. Univariate Analysis of Subject Characteristics and BMI Loss prior to Diagnosis

With respect to BMI loss prior to diagnosis, CD group experienced more BMI loss than UC group. BMI loss in the A1 subgroup was similar and it was only at older ages that the difference between CD and UC manifested. There was also a trend towards smoking being associated with an increased loss in BMI in CD compared to UC, whereas appendectomy history had little effect in UC but a protective effect in CD. In both disease groups, history of IBD surgery was associated with greater loss in BMI prior to diagnosis perhaps reflecting further the severity of the disease and lower BMI at presentation. Overall 43% of subjects with CD and 49% of subjects with UC experienced no significant weight loss prior to diagnosis defined as less than 5% BMI loss.

### 3.4. Multivariate Analysis of the Factors Associated with Weight Loss at Diagnosis in CD and UC

Table 3 displays regression analysis for CD and UC separately against subject and disease characteristics. Unadjusted and mutually adjusted analyses are presented. Montreal A1 was included as a variable to observe whether any significant trends seen in the full group analysis could have been affected by the small but potentially significant A1 population. In CD patients, younger age at diagnosis was associated with more weight loss in both univariate and multivariate analyses. Montreal A1 was significantly associated with weight loss on univariate analysis, but this was lost on applying multivariate analysis. Patients with history of appendectomy lost less BMI prior to diagnosis, whereas ileal disease patients experienced a greater reduction in BMI. Current smoker versus nonsmoker at time of diagnosis showed a trend towards greater reduction in BMI; however, it did not reach statistical significance. In UC patients, age at diagnosis, Montreal A1, and being ex-smoker were significant on univariate analysis, but this was lost after mutual adjustment.

## 4. Discussion

We have demonstrated that patients with IBD have a tendency to lose weight prior to diagnosis. This is not universal with nearly half of subjects not experiencing this. Underweight is only common at younger age of diagnosis. Weight loss was greater in subjects with CD than UC looking at all disease characteristics. In both CD and UC, younger age at diagnosis and history of previous surgery were the main factors associated with increased weight loss prior to diagnosis. In CD patients, ileal disease and absence of history of appendectomy were associated with an increased weight loss independently of age at diagnosis together with a trend towards smoking also being associated. The association of BMI loss with a history of surgery is likely to be a reflection of disease severity.

Weight loss in IBD could be contributed to through a number of different processes. Firstly, IBD is inflammatory in nature, resulting in a generalised catabolic state. Resting energy expenditure has been shown to be increased during acute flares in CD and proinflammatory cytokines exert an anorexic effect [5]. The inflammatory state in IBD has been linked to alterations in the levels of a number of metabolic hormones including leptin, adiponectin, and ghrelin which can affect satiety [6]. Secondly, these disease processes are associated with malabsorption of both macronutrients and micronutrients [7]. Finally, patients with IBD suffer an enhanced gastrocolic reflex and a variety of symptoms including pain often associated with ingestion of food that leads to avoidance of food [8].

Some of the above reasons may be useful in trying to explain one of the dominant findings of our study that CD patients lose more weight prior to diagnosis than UC patients. CD is associated with a more marked systemic inflammation than UC [9]. Another reason for the differences between UC and CD may be the way in which the two diseases present clinically and the subsequent challenges of diagnosis. The heterogenous nature of symptoms in CD may account for the delay in diagnosis, which in turn may lead to an increased perceived weight loss. Diagnostic lag has been shown to be significantly longer for CD over UC [10]. In a limited number of patients we had information on the age of the subject at their maximum preillness weight. For this analysis, only subjects with a maximum weight age up to 10 years before the diagnosis were considered. Eighty-four subjects with UC were at their maximum preillness weight 2.20(SEM 0.28) years versus 2.23(SEM 0.25) years old for CD; that is, there was no difference in our population. This would tend to discount delayed presentation as an explanation for the findings in our study population leaving the effect of systemic inflammation in CD more likely.

Younger age at diagnosis could be a manifestation of a more severe subtype of the overall disease process than the effect of growing itself. The pubertal growth spurt could be a potential confounding factor on BMI, notably in the Montreal A1 cohort, and normal ranges for BMI do vary in children. However growth retardation in pubertal patients with IBD is well described. Although univariate analysis showed a significant effect of being in the A1 category, this disappeared on multivariate analysis including adjustment for age at diagnosis. In our patient group, amongst the limited population with known age at maximum preillness weight, there was a significant positive correlation between increasing length of time and maximum preillness weight and age at diagnosis (correlation coefficient 0.267, *P* = 0.005), which tends to support the notion that severity of symptoms may outweigh any effect of younger age towards later diagnosis. Younger age at diagnosis in CD has been often cited as a predictor for poorer prognosis including chronic disabling disease [11]. The genetic component in younger patients may also be considered. Genes including NOD1/2 have been implicated in IBD pathogenesis. These have been shown to be more frequent in Montreal groups A1 compared to A3 subgroup [12], as well as being associated with more aggressive phenotypes [13].

In our CD population, ileal disease was associated with greater weight loss prior to diagnosis. Malabsorption is unlikely to solely explain the greater weight loss in subjects with ileal disease, given that ileal resection usually restores health. Atypical presentation leading to diagnostic delay may act as a factor to increased weight loss in this subgroup of patients. Ileal disease has been shown to be an independent risk factor for diagnostic delay in CD [12]. However, from our limited data on the age of maximum preillness weight, the time from maximum preillness weight to diagnosis was not greater in subjects with ileal disease and instead tended to be longer in subjects with colonic CD (time to diagnosis from maximum preillness weight: 1.90(SEM 0.46) years for ileal, 1.97(SEM 0.31) years for ileocolonic, and 3.47(SEM 0.77) years for colonic CD). Another potential explanation is that ileal disease is recognised with a more aggressive outcome. Patients with ileal disease require surgery more often [14], have lower fat mass, and have previously been shown to experience increased weight loss versus patients with disease localised elsewhere and normal controls [15]. In addition, patients with CD tend to suffer with abdominal pain more than UC patients [16], but it is not certain whether patients with pure ileal disease experience more pain than those with ileocolonic or colonic disease. However, given the propensity of pure ileal disease with stricture, this would not be surprising.

Smoking is associated with ileal CD and not with colonic CD [17]. In our CD cohort, smokers suffered higher rates of weight loss just falling short of formal statistical significance.

An intriguing finding from our data was the negative association of prediagnosis weight loss and history of appendectomy. Appendectomy Association with IBD has been repeatedly reported in the literature with a favourable effect on UC. Appendectomized patients have a lesser risk of developing UC. In the few appendectomized patients who developed UC, disease course was less severe, with a decreased need for colectomy compared to nonappendectomized patients [18]. In CD, the effect of previous appendectomy remains debated. Some series reported an increased risk of CD after appendectomy [19] and others did not [20]. Our results imply that history of appendectomy in CD patients provides a protective effect against weight loss prior to formal diagnosis. The findings suggest that appendectomy may be associated with a less aggressive disease phenotype. Unfortunately, we did not determine the details of appendectomy in our study, rather we just enquired into whether it had occurred or not.

There are some limitations to this study that merit discussion. A possible constraint may be selection bias, with the potential for patients with mild disease (i.e., not requiring regular follow-up in a secondary centre) having been underrepresented. The process of entering consecutive patients including many coming for routine annual review into the study hopefully minimised this. Our observation that recognised risk factors for CD versus UC such as smoking, appendectomy, and earlier age at diagnosis were associated in the expected way also adds validity to our findings.

BMI measurements, especially using self-reported weights, are an inferior measurement of true nutritional status as compared with more modern anthropometric techniques. However, the impracticalities of measuring parameters like hand grip strength, waist to hip ratios, and body fat percentage limit their use, especially in a retrospective study. Furthermore, we have already validated the use of self-reported weights in a small study, as discussed earlier.

## 5. Implications for Clinical Practice

This study has documented weight loss as a significant problem for many IBD patients at presentation, especially in younger age and CD with ileal involvement. However it is not universal particularly in older patients of whom nearly half do not experience any weight loss. To our knowledge, this is the first study to characterise weight loss prior to diagnosis in patients presenting with inflammatory bowel disease. Improved awareness of the presenting features of IBD should encourage wider use of screening tools and earlier investigative tests. Current tools used to help decide disease severity do not emphasize weight loss. The Harvey-Bradshaw Index does not incorporate any weight statistic into its calculation. The CD Activity Index does include a parameter for body mass, but its overall bearing on the total score appears limited.

## 6. Conclusion

Our study has characterised weight loss prior to diagnosis in patients with IBD. Earlier age and ileal located CD were strongly correlated with increasing weight loss prior to diagnosis, whereas history of appendectomy appears to confer some protection against weight loss. Weight loss monitoring and measurement of BMI should be assessed as simple and convenient methods for the assessment of IBD patients.

## Figures and Tables

**Table 1:** Disease characteristics of study population.

	Ulcerative colitis	Crohn's disease	All patients
Number of patients	257	237	494
Male : female (% female)	124 : 133 (51.8%)	117 : 120 (50.6%)	241 : 253 (51.2%)
Ethnic group			
Caucasian	191 (74.3%)	198 (83.5%)	389 (78.7%)
Asian	41 (15.9%)	29 (12.2%)	70 (14.2%)
Afro-Caribbean	11 (4.3%)	4 (1.7%)	15 (3.0%)
Other	14 (5.4%)	6 (2.5%)	20 (4.0%)
Age at diagnosis (years)			
Mean (st. error)	39.5 (1.05)	35.1 (1.09)	37.4 (0.76)
Range (median)	8–82 (35)	10–82 (30)	8–82 (33)
		*P* = **0.0041**	
Age group at diagnosis			
A1 < 17	7 (2.7%)	17 (7.2%) *P* = **0.034**	24 (4.9%)
A2 17–40	145 (56.4%)	142 (59.9%)	287 (58.1%)
A3 > 40	105 (40.9%)	78 (32.9%)	183 (37.0%)
Smoking status			
Nonsmoker	234 (91.1%)	182 (77.3%)	416 (84.2%)
Smoker	23 (8.9%)	55 (22.7%)	78 (15.8%)
		*P* < **0.0001**	
Appendectomy			
Negative	167 (88.8%)	139 (78.5%)	306 (84.3%)
Positive	21 (11.1%)	38 (21.5%), *P* = **0.0101**	59 (15.7%)
Unknown	69	60	129
Family history of IBD			
Positive	51 (19.8%)	45 (19.0%)	96 (19.4%)
Negative	206 (80.2%)	192 (81.0%)	398 (80.6%)
Family history of colorectal cancer			
Positive	29 (12.4%)	21 (9.8%)	50 (11.2%)
Negative	205 (87.6%)	193 (90.2%)	398 (88.8%)
Unknown	23	23	46
History of IBD surgery			
Positive	14 (8.0%)	88 (41.5%) *P* < **0.0001**	102 (26.4%)
Negative	161 (92.0%)	124 (58.5%)	285 (73.6%)
Unknown	82	25	107
Disease location			
Ileal	N/A	37 (17.5%)	N/A
Ileocolonic	N/A	124 (58.8%)	N/A
Colonic	N/A	50 (23.7%)	N/A
Unknown	N/A	26	N/A
Disease behaviour			
Inflammatory	N/A	139 (63.5%)	N/A
Stenotic	N/A	34 (15.6%)	N/A
Fistulating	N/A	46 (21.6%)	N/A
Unknown	N/A	18	N/A

All comparisons between UC and CD group were calculated with Fisher's exact test, with the exception of mean age at diagnosis where nonpaired *t*-test analysis was conducted. % calculated from patients with data only (i.e., unknown data excluded from % calculations).

**Table 2:** Mean BMI at diagnosis and % of BMI loss prior to diagnosis.

Characteristics	Mean BMI at diagnosis Mean BMI kg/m² (SE/range)		% BMI loss prior to diagnosis Mean BMI kg/m² (SE/range)	
	Ulcerative colitis	Crohn's disease	Ulcerative colitis	Crohn's disease
All patients	23.97 (0.33, 37.40)	23.68 (0.44, 43.10)	7.63 (0.59, 57.71)	9.76 (0.71, 57.99) *P* = 0.02^*^
Age at diagnosis				
A1	17.51 (1.42, 9.68)	16.28 (1.05, 14.72)	15.66 (7.10, 46.18)	14.25 (2.72, 36.98)
A2	22.74 (0.37, 32.26)	22.72 (0.52, 40.37)	8.50 (0.78, 56.36)	10.95 (1.00, 57.99)
A3	26.10 (0.53, 32.08)	27.03 (0.73, 28.75)	5.89 (0.80, 36.28)	6.58 (0.91, 40.27)
Sex				
Male	24.20 (0.43, 32.26)	23.07 (0.53, 29.71)	7.45 (0.89, 57.71)	9.22 (1.02, 56.02)
Female	23.76 (0.49, 35.26)	24.27 (0.70, 40.37)	7.79 (0.78, 44.84)	10.26 (1.00, 55.42)
Smoking				
Yes	22.75 (1.03, 23.31)	23.54 (0.76, 32.45)	7.45 (2.20, 44.78)	11.61 (1.70, 53.13)
No	24.09 (0.34, 37.40)	23.72 (0.53, 43.10)	7.64 (0.61, 57.71)	9.18 (0.77, 48.51)
Appendectomy				
Positive	26.35 (1.24, 23.60)	24.68 (0.92, 20.49)	7.46 (2.30, 46.20)	7.46 (1.73, 43.17)
Negative	24.15 (0.43, 37.40)	23.29 (0.61, 43.10)	7.05 (0.74, 56.36)	10.98 (0.95, 55.42)
Family history of IBD				
Positive	22.26 (0.65, 26.32)	23.99 (0.95, 24.83)	9.91 (1.58, 48.81)	9.21 (1.32, 33.68)
Negative	24.40 (0.37, 35.71)	23.60 (0.50, 43.10)	7.05 (0.61, 53.66)	9.89 (0.82, 57.99)
Family history of CRC				
Positive	21.56 (0.55, 12.42)	24.14 (1.83, 36.93)	8.28 (2.05, 50.44)	14.49 (2.81, 37.11)
Negative	24.40 (0.38, 37.40)	23.35 (0.46, 41.54)	7.45 (0.64, 57.71)	9.90 (0.79, 57.99)
History of IBD surgery				
Positive	20.95 (0.92, 1.24)	22.27 (0.67, 41.54)	9.61 (2.39, 29.63)	11.94 (1.33, 53.13)
Negative	24.11 (0.39, 35.71)	24.70 (0.64, 41.88)	7.55 (0.73, 55.87)	8.44 (0.89, 46.81)
Disease location				
Ileal	N/A	23.86 (1.07, 26.46)	N/A	11.31 (1.99, 36.07)
Ileocolonic	N/A	23.35 (0.61, 43.10)	N/A	10.46 (1.00, 57.99)
Colonic	N/A	24.27 (1.03, 33.63)	N/A	8.77 (1.62, 55.56)
Behaviour				
Inflammatory	N/A	23.88 (0.59, 43.10)	N/A	8.92 (0.88, 48.05)
Stenotic	N/A	24.01 (1.05, 26.49)	N/A	9.75 (1.82, 43.59)
Fistulating	N/A	23.00 (1.01, 37.01)	N/A	12.44 (1.84, 53.13)

^*^%BMI loss, UC versus CD.

**Table 3:** Regression analysis of %BMI change prior to diagnosis in CD/UC subgroups.

	Ulcerative colitis		Crohn's disease	
	Unadjusted univariate analysis	Multivariate analysis	Unadjusted univariate analysis	Multivariate analysis
Age at diagnosis (per year)	−0.105	−0.14	−0.172	−0.161
	(−0.179–−0.031)	(−0.035–−0.0008)	(−0.258–−0.087)	(−0.25–−0.067)
	*P* = **0.005**	*P* = 0.2150	*P* = **0.0001**	*P* = **0.008**

Montreal A1	9.066	1.445	6.018	2.841
	(1.285–16.847)	(−0.735–3.625)	(0.629–11.406)	(−2.951–8.633)
	*P* = **0.0226**	*P* = 0.2150	*P* = **0.0288**	*P* = 0.3348

Sex (male versus female)	−0.827	−0.279	1.314	1.053
	(−3.331–1.677)	(−0.972–0.414)	(−1.616–4.244)	(−1.806–3.912)
	*P* = 0.5160	*P* = 0.4287	*P* = 0.3780	

Smoking at time of diagnosis (versus nonsmoking)^*^	−0.361	−0.429	2.778	3.426
	(−1.590–0.869)	(−1.670–0.811)	(−0.930–6486)	(−0.232–7.058)
	*P* = 0.5641	*P* = 0.4963	*P* = 0.1413	*P* = 0.0663

Ex-smoker (versus nonsmokers)	−0.770	−0.717	0.135	2.324
	(−1.477–−0.062)	(−1.455–0.021)	(−3.308–3.578)	(−1.152–5.800)
	*P* = **0.0331**	*P* = **0.0570**	*P* = 0.9384	*P* = 0.1891

History of appendectomy (versus none appendectomy)^**^	0.364	0.503	−3.306	−4.170
	(−0.902–1.635)	(−0.785–1.791)	(−7.390–0.778)	(−8.219–−0.122)
	*P* = 0.5738	*P* = 0.4424	*P* = 0.1121	*P* = **0.0436**

Ileal disease (versus non ileal disease)	N/A	N/A	3.534	3.991
			(−0.457–7.525)	(0.068–7.913)
				*P* = **0.0462**

All variables are mutually adjusted for each other. ^*^adjusted for ex-smokers. ^**^adjusted for those where appendectomy data are missing.
